# Phenotypic Matching Without Genetic Correlation in Dimorphic Legs of Bulb Mites

**DOI:** 10.1002/ece3.72791

**Published:** 2026-01-15

**Authors:** Diego Solano‐Brenes, Kyana N. Pike, Joseph L. Tomkins, Glauco Machado, Bruno A. Buzatto

**Affiliations:** ^1^ Programa de Pós‐graduação em Ecologia, Instituto de Biociências Universidade de São Paulo São Paulo Brazil; ^2^ Health & Biosecurity Commonwealth Scientific and Industrial Research Organisation (CSIRO) Townsville Queensland Australia; ^3^ Centre for Evolutionary Biology, School of Biological Sciences The University of Western Australia Perth Western Australia Australia; ^4^ LAGE do Departamento de Ecologia, Instituto de Biociências Universidade de São Paulo São Paulo Brazil; ^5^ College of Science and Engineering Flinders University Bedford Park South Australia Australia

**Keywords:** condition‐dependence, intrasexual competition, intrasexual dimorphism, phenotypic correlation, sexual selection, weapon allometry

## Abstract

The evolution of exaggerated structures used as weapons in male–male contests often drives correlated changes in traits that compensate for their costs or enhance their benefits. Given their interdependence, weapons and compensatory traits are expected to evolve with some degree of genetic integration. The bulb mite *Rhizoglyphus echinopus*, a male‐dimorphic species, offers an opportunity to examine this question. In this species, the third legs are dimorphic in width, functioning as a weapon, while the fourth legs are dimorphic in length, potentially serving as a compensatory trait. Fighter males, identified by their thicker third legs, also tend to have longer fourth legs than scramblers. Here, we first tested whether body size and the width of the third legs predict contest success, and whether the length of the fourth legs also contributes to fighting ability. We then evaluate phenotypic and genetic integration between these traits. Behavioral trials showed that variation in body size, third leg width, and fourth leg length did not explain contest outcomes. Moreover, although males with thicker third legs tended to have longer fourth legs, quantitative genetic analyses revealed that the traits are phenotypically, but not genetically, correlated. This suggests that their phenotypic match likely results from shared environmental thresholds rather than genetic integration. Overall, our findings emphasize the need to move beyond phenotypic correlations when evaluating the evolutionary role of compensatory traits, as such correlations alone may mislead interpretations of their adaptive significance.

## Graphical abstract

We tested for male dimorphism in the weapon‐like third legs and putative compensatory fourth legs of the bulb mite *Rhizoglyphus echinopus*. Although males with thicker third legs tended to have longer fourth legs, neither trait predicted fighting success, and the correlation was phenotypic but not genetic. Our results highlight that phenotypic matches between weapon and compensatory traits can arise without genetic integration, cautioning against inferring evolutionary mechanisms from correlations alone.

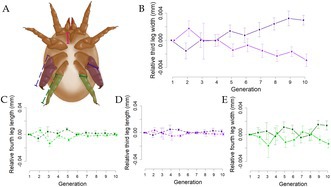

## 1 Introduction

Strong intrasexual competition, particularly among males, often drives the evolution of exaggerated structures known as weapons, which are used in contests for mating opportunities (Emlen 2008; Rico‐Guevara and Hurme 2019). The effectiveness of these weapons in inflicting damage or signaling strength typically determines the winner of the contest (Judge and Bonanno 2008; Yoshino et al. 2011; Yasuda and Koga 2016; Emberts et al. 2021; reviewed in Palaoro and Peixoto 2022). For instance, male rhinoceros beetles possess prominent horns on their head and thorax, which they use during contests. The shape of these horns is species‐specific, optimizing their performance according to the males' fighting style (McCullough et al. 2014). Despite the high costs of producing and maintaining exaggerated weapons (Emlen 2001; Painting and Holwell 2013; Goyens et al. 2015; Somjee et al. 2018; O'Brien et al. 2019), these structures remain adaptive, as winning contests provides significant fitness advantages by granting exclusive access to females or mating sites (Kelly 2006; Buzatto and Machado 2008; Yoshino et al. 2011; Painting and Holwell 2014).

The costs associated with exaggerated weapons in males can be mitigated through the evolution of additional modified structures, known as *compensatory traits*, which are distinct from the weapons themselves (Tomkins et al. 2005). These traits are thought to be adaptations that reduce the costs of exaggerated weapons in terms of survival, locomotion, or energetic performance. For example, in several species of bovids, males use their horns as weapons to stab, ram, wrestle, or fence with opponents (Vander Linden and Dumont 2019). In these species, the morphology of the neck vertebrae—structures that are not part of the weapons themselves—varies according to fighting style, likely representing adaptations to absorb impact during contests (Vander Linden and Dumont 2019).

While compensatory traits are thought to evolve as adaptations that offset functional challenges imposed by weapons, other modifications may instead enhance the effectiveness of weapons. In this context, the benefits of weapons can be further amplified by the evolution of changes in structures unrelated to the weapons themselves. These structures, referred to as *supportive traits*, have been documented in some insects with exaggerated weapons, functioning to increase the stability and efficiency of weapons in contests (Tatsuta et al. 2004; Okada and Miyatake 2009; Okada et al. 2012). For instance, in the beetle *Gnatocerus cornutus* (Tenebrionidae), males engage in contests using enlarged mandibles. Males with larger mandibles also have larger heads, prothoraxes, and forelegs, which may provide structural support for the increased force exerted during contests (Okada and Miyatake 2009). Notably, compensatory and supportive functions are not mutually exclusive, and the same trait may serve both roles (Tomkins et al. 2005).

The correlated evolution between exaggerated weapons and other morphological traits can result from genetic correlations (Cheverud 1996; Wagner 1996; Tomkins et al. 2005). Such correlations may be shaped by selective pressures—whether natural or sexual selection—that favor the modular expression of traits originally unrelated (Melo et al. 2016). In the beetle *G. cornutus* , artificial selection experiments demonstrated integration between mandible size and other contest‐related traits. Males with larger mandibles developed proportionally larger compensatory or supportive traits, whereas males with smaller mandibles developed correspondingly smaller traits (Okada and Miyatake 2009). Although phenotypic or genetic correlations between weapons and other contest‐related traits have been suggested in several species (Tomkins et al. 2005; Painting and Holwell 2013; Ito et al. 2017; Miller et al. 2024), studies directly testing these correlations using quantitative genetics remain scarce (Okada and Miyatake 2009). Clarifying the genetic bases of these correlations is essential to determine whether the coexpression of traits arises from shared developmental pathways or selective pressures favoring phenotypic integration. Moreover, understanding how the evolution of exaggerated weapons influences male body shape can provide valuable insights into the origins of morphological diversity.

In *Rhizoglyphus* bulb mites (Acaridae), males exhibit dimorphism in the width of their third pair of legs (Woodring 1969). Fighter males, characterized by thicker third legs, use these appendages as weapons during male–male contests (Radwan 2009). In contrast, scramblers—with thinner third legs—avoid fighting altogether and rely on sneak copulation as their reproductive tactic (Radwan 2009). Unlike the stereotyped face‐to‐face encounters observed in many other taxa (Emlen 2014), contests among bulb mites appear opportunistic. In *R. robini*, for instance, when a fighter encounters another male, it climbs onto its opponent's body and uses the claws of its third legs to pierce and kill the rival (Radwan et al. 2000). However, as in most animal species with exaggerated weapons, the specific traits that determine contest success in bulb mites remain unclear, and the role of the enlarged third legs has yet to be directly tested. Furthermore, the potential contribution of other legs as compensatory or supportive traits has never been investigated in this well‐studied arachnid group. *Rhizoglyphus echinopus*, in particular, represents a tractable system to address these gaps, as male–male contests can be easily staged in the laboratory and the morphological traits influencing their outcomes formally investigated.

*Rhizoglyphus* bulb mites also provide an excellent study system for investigating phenotypic integration through artificial selection experiments, as the genetic bases of both sexual and intrasexual dimorphism have already been examined. In *R. echinopus*, the developmental threshold that determines male morph expression has been shown to be heritable (Buzatto et al. 2012). In addition, genetic correlations have been reported both between male morphs and between sexes in this species. For example, Pike et al. (2017) found a strong genetic correlation in the width of the third pair of legs between fighter and scrambler males. Similarly, bidirectional selection on this trait in fighter males—favoring either thicker or thinner legs—produced parallel evolutionary responses in scrambler males and females (Buzatto et al. 2018). Despite these advances, it is still unclear whether, and in what ways, selection on the third legs of fighter males might influence other traits.

In this study, we provide the first formal evidence that the third and fourth pairs of legs in bulb mites are male‐dimorphic. Whereas the third legs are dimorphic in *width*, the fourth legs are dimorphic in *length*, and we propose that the latter may function as a compensatory or supportive trait. To assess the functional role of these traits, we conducted an experiment testing the effects of body size and the two leg traits on male–male contest outcomes. Building on a recent meta‐analysis showing that winners tend to have larger bodies and weapons than losers (Palaoro and Peixoto 2022), we predicted that both body size and third leg width would increase contest success. Moreover, if the fourth legs serve as a compensatory or supportive trait, we expected their length to also contribute to contest outcomes. Finally, using two complementary approaches, a paternal half‐sibling design and a bidirectional artificial selection experiment, we investigated the genetic correlation between third leg width and fourth leg length. Given their simultaneous exaggeration in fighter males, we expected a strong genetic correlation between them. Accordingly, artificial selection on the width of the third legs should drive a correlated evolutionary response in the length of the fourth legs.

## 2 Materials and Methods

### 2.1 Leg Dimorphism

**FIGURE 1:**
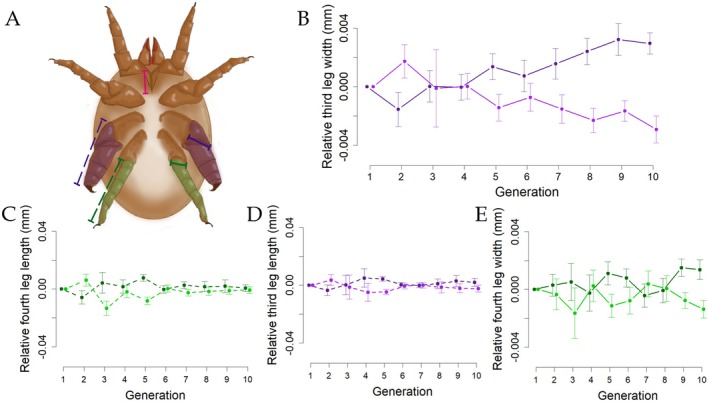
(A) Schematic representation of a fighter male of the bulb mite *Rhizoglyphus echinopus* showing the length of the anterior coxal suture (pink line) and the leg traits analyzed: Length (dashed lines) and width (solid lines) of the third (purple) and fourth (green) pairs of legs. (B) Results of the bidirectional artificial selection over 10 generations on the width of the third pair of legs (previously published in Buzatto et al. 2018). (C–E) Effects of this selection on other leg traits (this study). Darker lines represent the upward selection lines, while lighter lines represent the downward selection lines. Dots indicate the mean values, and error bars represent the standard error for each trait in each generation.

To test for male dimorphism in the width and length of both the third and fourth pairs of legs in *Rhizoglyphus echinopus*, we used measurements from 221 scramblers and 221 fighters originating from the half‐sibling design experiment (see below). We mounted these males on slides and used a microscope (Leica M60) to measure the width and length of both the third and fourth pairs of legs. As a proxy for body size, we also measured the length of the anterior coxae suture (Figure 1A) as described in Pike et al. (2017). Next, we fitted mixture models to the distributions of third and fourth leg measurements, comparing a single skew‐normal distribution with a mixture of two skew‐normal distributions (for each trait) using the Akaike information criterion (AIC). Additionally, we evaluated the allometry of the fourth pair of legs using a linear model with the anterior coxae suture length, as well as its interaction with male morph (based on the width of the third pair of legs), as independent variables. We also fitted linear models to examine how the width of the third pair of legs explains the length of the fourth pair of legs within morphs. To fit the mixture models, we used the *mixsmsn* library (Prates et al. 2013), and to fit the allometries of the fourth pair of legs and its relationship with the third pair of legs, we used the *stats* package in the software R (R Core Team 2024).

### 2.2 Fighter Contests Experiment

To assess the effects of leg and body size on the probability of winning a contest, we randomly paired unrelated fighter males (*n* = 25 pairs) in a plastic container (diameter = 30 mm) with a virgin female for 24 h. At the end of this period, we recorded the identity of the surviving male as the winner and the deceased male as the loser. The males were then mounted on microscope slides, and measurements were taken of the anterior coxae suture length, the width of one of their third legs, and the length of one of their fourth legs. To prevent measurement bias, each male was assigned a non‐informative code to ensure “blind” measurements. These trials were conducted between 2018 and 2019.

To identify the characteristics that determine the winner of the contests, we selected the best model from two sets of models: one using the relative sizes of traits between opponents as predictor variables, and the other using their absolute sizes. Relative sizes were calculated for both males in each pair by subtracting the trait value of one from the other (e.g., for male 1 in pair *n* = trait_male 1_—trait_male 2_; for male 2 = trait_male 2_—trait_male 1_). We first fitted global generalized linear mixed models (GLMM), for both the absolute and relative sizes, using the width of the third pair of legs, the length of the fourth pair of legs, the anterior coxae suture length (i.e., body size), as well as all possible two‐way interactions and the three‐way interaction between these variables as predictors. The response variable was the contest outcome for each of the males (1 for winners and 0 for losers), and the identity of the contest was modeled as a random effect. We assumed a binomial error distribution and used a logit link function for all models. Then, we performed a model selection between the global model and the subset of simpler models derived from it. We performed the model selection using the bias‐corrected version of the Akaike information criterion to small samples (AIC_c_) to identify the model that best explained the probability of winning. Analyses were conducted using the packages *lme4* (Bates et al. 2015) and *MuMIn* (Bartoń 2023) in the software R (R Core Team 2024).

### 2.3 Half‐Sibling Design Experiment

The rearing conditions, selection protocol, and genetic analyses for this experiment are described in detail in Pike et al. (2017). Briefly, the first generation (F0) was established by pairing 28 virgin fighter males sequentially with two virgin females each, resulting in 56 initial families, of which 44 produced offspring. Each pair was allowed to breed for 4 days, after which the females were isolated to lay their eggs. Once the eggs hatched, 30 larvae were randomly selected and individually isolated (F1). The larvae were fed *ad libitum* with dried baker's yeast until they reached adulthood. For the next generation, 32 fighter males from 22 families were selected from the F1. In this generation, males were paired simultaneously rather than sequentially as in the previous generation, with two virgin females each. Paternal individuals and four fighter siblings from each generation were mounted on microscope slides for body size and dimorphic legs measurements (*n* = 221).

For the analyses, we estimated the genetic correlation between the width of the third pair of legs and the length of the fourth pair of legs using a bivariate animal model. The model included the mean of each leg trait and their interaction as fixed effects, with the additive genetic effect of each individual treated as a random effect. Genetic correlations were calculated as the covariance between the traits divided by the square root of the product of their respective variance components (Lynch and Walsh 1998). To compare the genetic correlations, we used 84% confidence intervals for each estimate. The models were implemented in the software R using the *ASReml‐R* package with restricted maximum likelihood estimation (Butler et al. 2009).

### 2.4 Bidirectional Artificial Selection Experiment

From May 2016 to October 2017, we conducted a bidirectional, morph‐specific artificial selection experiment on the third pair of legs in fighter males over 10 generations. The rearing conditions and selection protocol are described in detail in Buzatto et al. (2018). Briefly, we began with 75 families, each consisting of one virgin fighter male and three virgin females, all reared in isolation. These families were divided into three replicate lines (A, B, and C), with 25 families per line. After 2–4 days of mating, males were mounted on microscope slides, and the width of the right third leg and the anterior coxae suture length were measured, following Pike et al. (2017).

Using Type I linear models, we evaluated the relationship between these traits and selected the five males with the largest (up lines) or smallest (down lines) residuals to establish six replicate lines: A‐up, A‐down, B‐up, B‐down, C‐up, and C‐down. Forty offspring from each selected male were reared in isolation to produce virgin adults for the next generation. These adults were sexed and scored for morph, providing data on sex and morph ratio. From these individuals, 20 fighters per line were paired with two unrelated females from the same line. This selection protocol was followed for nine additional generations, with separate linear models for each line and five males selected from a pool of 20 per generation.

Throughout the experiment, all individuals were reared in isolation in cylindrical glass vials (diameter = 10 mm, height = 14 mm) and mated in plastic tubes (diameter = 25 mm, height = 14 mm). Both the individual vials and mating tubes had a 3‐mm thick plaster‐of‐Paris base, kept damp by placing them on moist filter paper on Petri dishes. All vials and tubes were stored in cylindrical plastic containers (diameter = 76 mm, height = 40 mm) inside dark incubators set at 22°C.

The results of the selection response on the width of the third pair of legs of fighter males, as well as the correlated response in the same legs of scrambler males and females, have been previously reported in Buzatto et al. (2018) (Figure 1B). In this study, we focus on the coevolution of the width of the third pair of legs with additional traits, with particular emphasis on another male dimorphic trait: the length of the fourth pair of legs, which is hypothesized to have a supportive or compensatory function.

To track the effect of artificial selection on the width of the third pair of legs and the length and width of the fourth pair of legs, as well as the length of the third pair in fighters, we first calculated the relative size of these three leg traits from the sixth generation onward—a point at which the selection lines for third‐leg width were clearly differentiated (figure 1B; see also Buzatto et al. 2018). To do that, we fitted separate linear models to the relationship between the leg measurement and the anterior coxae suture length in each generation. Next, we extracted the models' residuals from both directions of selection (up and down lines), allowing a direct comparison of the up and down lines in each generation while removing environmental effects. Then, we fitted linear mixed effects models using the relative size of the legs as response variables and line ID as a random effect. As predictor variables, we used generation, selection direction (up and down), and their interaction. We fitted a set of five models with every possible combination of the predictors and compared them using AIC_c_. Analyses were conducted using the *MuMIn* package (Bartoń 2023) in the software R (R Core Team 2024).

## 3 Results

### 3.1 Leg Dimorphism

**FIGURE 2:**
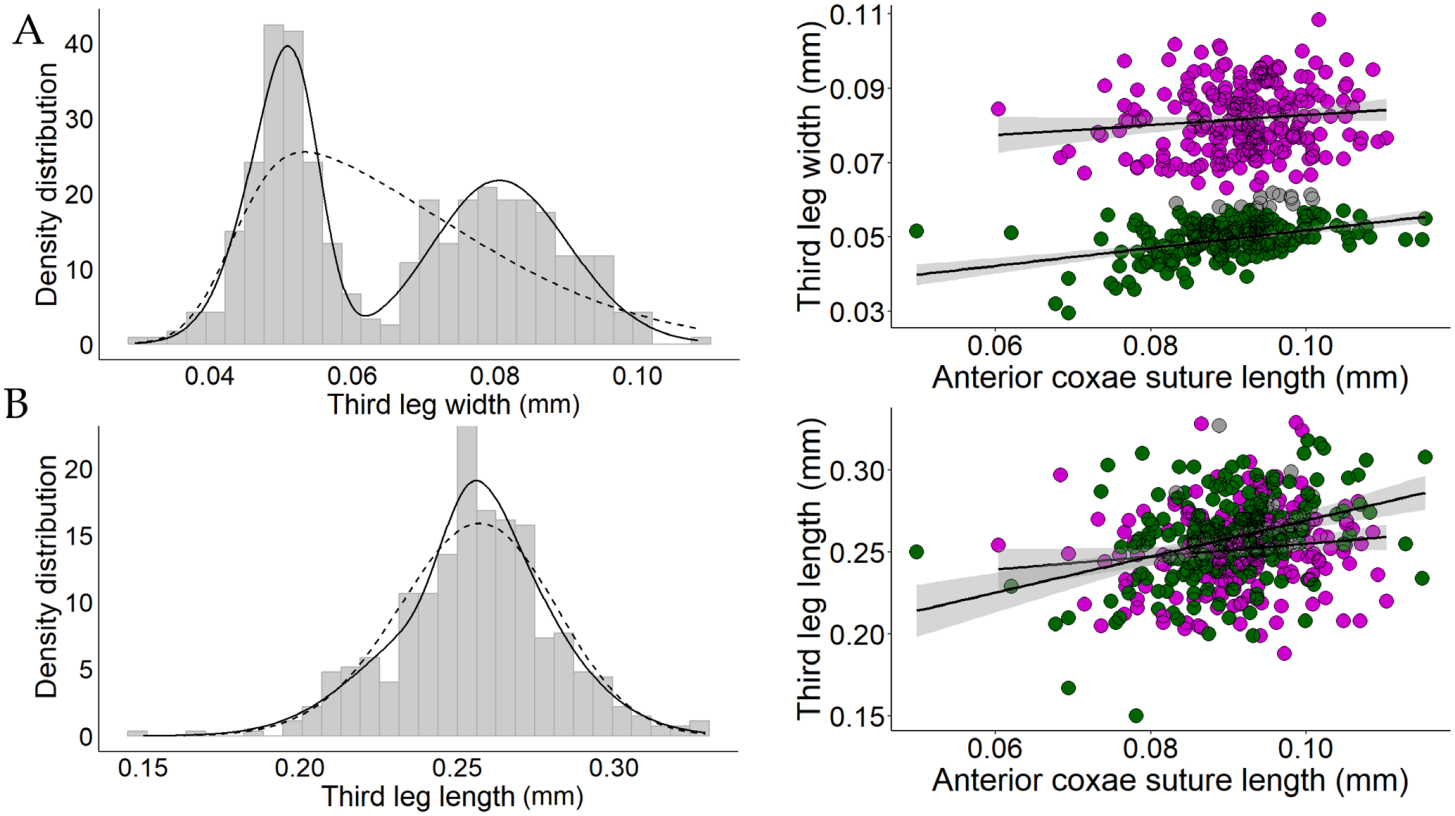
Density distribution and allometries of third leg width (A) and length (B) of fighters (purple), scramblers (green), and uncategorized individuals (gray) of the bulb mite *Rhizoglyphus echinopus*. The continuous line in the density distribution represents the density curve according to the model with a mixture of two distributions, while the dashed line represents the density curve according to the model with just one distribution. The lines in the allometry plots represent the estimated relationship between leg length and a proxy for body size (anterior coxae suture length), with the shaded area indicating the 95% confidence interval. The male morphs were determined based on the width of the third pair of legs.

**FIGURE 3:**
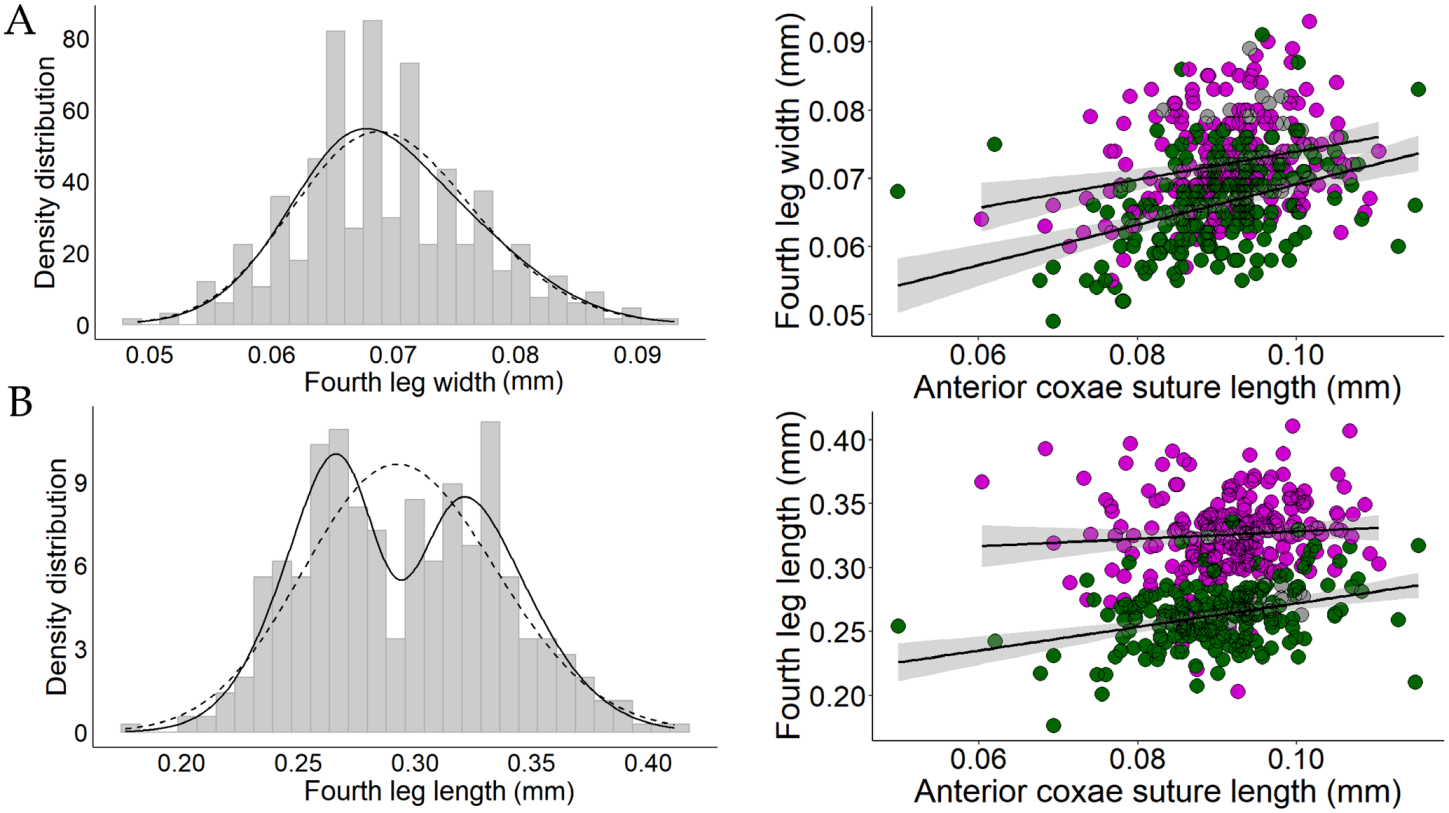
Density distribution and allometries of fourth leg width (A) and length (B) of fighters (purple), scramblers (green), and uncategorized individuals (gray) of the bulb mite *Rhizoglyphus echinopus*. The continuous line in the density distribution represents the density curve according to the model with a mixture of two distributions, while the dashed line represents the density curve according to the model with only one distribution. The lines in the allometry plots represent the estimated relationship between leg length and a proxy for body size (anterior coxae suture length), with the shaded area indicating the 95% confidence interval. The male morphs were determined based on the width of the third pair of legs.

The mixture of two skew‐normal distributions best fit the width of the third pair of legs (Figure 2A; ΔAIC_*c*_ = 168.36), classifying 97% of the males (*n* = 425) as either fighters or scramblers (using a minimal probability of 90% for membership in either morph). Similarly, the mixture of two skew‐normal distributions best fit the length of the fourth pair of legs (Figure 3B; ΔAIC_*c*_ = 26.10), strongly suggesting intrasexual dimorphism for this trait. In this case, a total of 85% (*n* = 377) of the males were classified into one of the two morphs (with at least 90% probability of belonging to that morph). In contrast, in both the length of the third leg (Figure 2B) and the width of the fourth leg (Figure 3A), the best fit model was the one with a single skew‐normal distribution.

The fourth pair of legs was longer in fighters than in scramblers, but its length did not change with body size in either morph (Figure 3B and Table S1). Among males with a 90% or higher probability of belonging to a given morph (based on the third pair of legs), 79% (*n* = 367) exhibited matching morph classification when considering the fourth pair of legs (i.e., fighters with longer fourth legs and scramblers with shorter fourth legs). In contrast, only 5% (*n* = 13) of fighters had fourth legs as short as those of scramblers, and 5% (*n* = 15) of scramblers had fourth legs as long as those of fighters. Within morphs, the width of the third pair of legs explained approximately 10% of the variation in fourth leg length among fighters (*p* < 0.001) and 22% among scramblers (*p* < 0.001).

### 3.2 Fighter Contests Experiment

For both sets of models (those using relative leg sizes and those using absolute leg sizes), the null models provided a better fit than models incorporating leg traits and body size in explaining the probability of winning a contest (Table 1).

**TABLE 1:** Top 10 models used to predict the probability of a fighter male of the bulb mite *Rhizoglyphus echinopus* winning a contest based on both the absolute and relative size of its legs. We used as predictor variables the width of the third pair of legs (3LW), the length of the fourth pair of legs (4LL), and the length of the anterior coxae suture as a proxy for body size (BS). For each model, we show the number of parameters (k), the Akaike information criterion for small samples (AIC_c_), the difference between the best model and the focal model (ΔAIC_c_), and the AIC_c_ weight (Weight).

Model	k	AIC_c_	ΔAIC_c_	Weight
**Absolute size**				
Null	1	73.57	0.00	0.36
4LL	2	74.96	1.39	0.18
3LW	2	75.81	2.24	0.12
BS	2	75.83	2.26	0.12
3LW + 4LL	3	77.22	3.65	0.06
4LL + BS	3	77.33	3.76	0.05
3LW + BS	3	78.16	4.59	0.04
3LW + 4LL + (3LW × 4LL)	4	79.35	5.78	0.02
3LW + 4LL + BS	4	79.67	6.10	0.02
4LL + BS + (4LL × BS)	4	79.76	6.19	0.02
**Relative size**				
Null	1	73.57	0.00	0.30
4LL	2	74.09	0.51	0.23
3LW	2	75.79	2.22	0.10
BS	2	76.20	2.25	0.10
3LW + 4LL	3	76.45	2.63	0.08
4LL + BS	3	78.11	2.88	0.07
3LW + BS	3	78.65	4.54	0.03
3LW + 4LL + BS	4	78.92	5.08	0.02
3LW + 4LL + (3LW × 4LL)	4	80.59	5.10	0.02
4LL + BS + (4LL × BS)	4	81.24	5.35	0.02

### 3.3 Half‐Sibling Design Experiment

We found no significant genetic correlation between the width of the third pair of legs and the length of the fourth pair of legs in fighters (Table 2). However, the widths of the third and fourth legs and the lengths of the third and fourth legs were highly genetically correlated (Table 2). Additionally, both the length and width of the third and fourth leg pairs showed significant yet moderate heritabilities (Table S2).

**TABLE 2:** Genetic correlations between the length and width of the third and fourth pairs of legs in fighter males of the bulb mite *Rhizoglyphus echinopus*. We report the genetic correlation for each pair of traits along with their standard error. Values in parentheses represent the probabilities that the correlations are equal to zero.

	Third leg width	Third leg length	Fourth leg width
Third leg width	—	—	—
Third leg length	0.43 ± 0.24 (0.074)	—	—
Fourth leg width	0.83 ± 0.13 (< 0.001)	0.39 ± 0.26 (0.134)	—
Fourth leg length	0.41 ± 0.28 (0.144)	0.80 ± 0.17 (< 0.001)	0.38 ± 0.29 (0.191)

### 3.4 Bidirectional Artificial Selection Experiment

The artificial selection experiment showed that only one of the traits of the fighters' legs responded to the selection originally targeting the width of the third pair of legs (Table 3, Figure 1C–E). Based on model selection, there were three equally parsimonious models for the evolution of the relative width of the fourth pair of legs. The best‐fitting model included only the direction of selection as a predictor variable, followed by a model with the interaction between direction of selection and generation, and another with the additive effects of direction of selection and generation (Table 3). In contrast, for both the length of the third and fourth pairs of legs, the best‐fitting models were the null models (Table 3).

**TABLE 3:** Model selection of linear mixed models used to predict the evolution of leg traits in fighters of the bulb mite *Rhizoglyphus echinopus*. We used as predictor variables the direction of selection and the generation of the bidirectional selection on the width of the third pair of legs of fighters. For each model, we show the number of parameters (k), the Akaike information criterion for small samples (AIC_c_), the difference between the best model and the focal model (Δ), and the AIC_c_ weight (Weight).

Model	k	AIC_c_	ΔAIC_c_	Weight
**Fourth** **leg width**				
Direction	4	−4297.50	0.00	0.54
Direction * Generation	6	−4295.75	1.75	0.22
Direction + Generation	5	−4295.71	1.79	0.22
Null	3	−4290.06	7.43	0.01
Generation	4	−4288.04	9.45	0.00
**Fourth leg length**				
Null	3	−2793.98	0.00	0.51
Direction	4	−2792.08	1.90	0.20
Generation	4	−2791.95	2.03	0.19
Direction + Generation	5	−2790.05	3.93	0.07
Direction * Generation	6	−2788.08	5.90	0.03
**Third** **leg length**				
Null	3	−2328.95	0.00	0.42
Direction	4	−2328.11	0.85	0.27
Generation	4	−2326.92	2.03	0.15
Direction + Generation	5	−2326.06	2.89	0.10
Direction * Generation	6	−2325.03	3.92	0.06

## 4 Discussion

Here, we provide the first formal test of intrasexual dimorphism in both the third and fourth pairs of legs in the bulb mite *Rhizoglyphus echinopus*. We show that when male morphs are defined based on the width of the third leg, fighters tend to have longer fourth legs than scramblers. We also tested whether third leg width, fourth leg length, and body size influence the outcome of male contests but found no evidence for an effect of leg morphology or body size on the probability of winning a contest. Regarding the hypothesis that the two male dimorphic traits (third leg width and fourth leg length) are phenotypically integrated, we found no evidence for a genetic correlation between these traits. In contrast, we found strong genetic correlations between the widths of the third and fourth legs and also the lengths of the third and fourth legs. Consistent with these findings, in the bidirectional selection experiment on the width of the third pair of legs, we observed correlated evolution on the width of the fourth pair of legs, but no changes in the length of either pair of legs.

We found no evidence of the influence of weapon size on fighting success, nor of a compensatory function of the fourth pair of legs during the contests. Although the number of contests is limited, the complete lack of pattern in the results suggests a genuine absence of effect of the tested leg traits on fight outcomes. Similar results have been observed in a meta‐analysis of several species: when male traits serve as “pure weapons” (sensu Eberhard et al. 2018) in physical contests, differences in trait size between winners and losers tend to be small or nonexistent (Palaoro and Peixoto 2022). In contrast, when male traits serve as “threat devices” (sensu Eberhard et al. 2018) to signal size or strength, differences in trait size between males usually determine the contest winner (Palaoro and Peixoto 2022). Unlike spider mites, in which males use their legs to push and grapple rivals, likely as part of an evaluation ritual (Potter et al. 1976), there is no evidence that bulb mites use either the third or fourth pair of legs as threat devices. This suggests that the only function of the third pair of legs in bulb mites is to inflict damage on the opponent, and as would be expected from a pure weapon, its efficiency does not vary with size among fighters.

If the legs in bulb mites are not used as threat devices, our results also support the hypothesis that these structures do not experience strong selective pressures favoring an increase in size, as directional selection on contest‐related traits is expected to act primarily on threat devices (Eberhard et al. 2018). As shown in Pike et al. (2017) and in our study, the width of the third pair of legs does not grow allometrically with body size. This finding is consistent with the absence of an effect of weapon size on contest outcome. However, unlike our results, Rhebergen et al. (2022) report a positive relationship between the width of the third pair of legs and the size of the quiescent tritonymph in *R. robini* in both morphs (stronger in fighters). A crucial difference between our data and that study is that we used an adult morphological trait (the anterior coxal suture) as our proxy for body size, rather than quiescent tritonymph size. The latter would be much more appropriate when inferring the cue that triggers development into the fighter morph (Bonduriansky 2007; Zinna et al. 2018). However, we used the former to remain consistent with our previous experiments designed to measure genetic correlations and responses to artificial selection. In those approaches, it is unfeasible to measure quiescent tritonymph size for the large number of individuals required, especially since only a small fraction of tritonymphs develop into fighters. It is therefore possible that we have not captured body size variation accurately in our experiments. Nonetheless, when it comes to weapon size, it seems that at least in *R. echinopus*, having fighter legs is sufficient for a male to win a contest, and the variation in leg size among fighters is not linked to contest outcome.

We found a phenotypic match between the third and fourth leg morphs in our study, but no evidence of a genetic correlation between traits underlying these dimorphisms. This morphological match may be explained by the conditional expression of the leg morphs in response to similar environmental cues (Roff 1996). According to the environmental threshold model, the expression of alternative phenotypes is influenced by an environmental cue, such as body size, so that at a particular point in development, individuals below a threshold value of size express one phenotype, whereas those above the threshold express another (Tomkins and Hazel 2007). Although many studies consider body size itself to be the environmental cue, it is important to note that body size results from a combination of truly environmental cues (e.g., food availability, population density) and its genetic basis (Tomkins and Hazel 2007). In bulb mites, specifically, the exaggeration of the third legs is indeed correlated with nymphal body size before adulthood (Buzatto et al. 2012). The expression of thicker third legs is also influenced by food quantity (Rhebergen et al. 2022), population density (Radwan 2001), and habitat complexity (Tomkins et al. 2011). To date, no information exists on whether these cues also affect the elongation of the fourth pair of legs. However, the patterns we observed could be explained by the expression of these two dimorphic leg traits being synchronized by shared environmental cues, without necessarily requiring a strong genetic correlation.

Despite the absence of an effect of the length of the fourth pair of legs on contest outcome, these legs could still act as a compensatory trait. For instance, long fourth legs could reduce the mobility costs associated with carrying a third thicker and probably heavier pair of legs. It has been demonstrated that fighters paid more costs on mobility than scramblers in structurally complex environments (Tomkins et al. 2011). This adaptive hypothesis for the phenotypic matching of the two pairs of legs suggests similar thresholds determine their morphs. Given that legs are not genetically correlated, the thresholds for the expression of their dimorphisms must show some degree of coordination. Therefore, individuals “mismatched” for the dimorphisms (i.e., fighters with short fourth legs and scramblers with long fourth legs) should be negatively selected, as these fighters are not compensating the cost of the large third pair of legs, and these scramblers are investing more resources in a useless elongation of the fourth legs. As shown in our results, males with mismatched leg morphs are rare. Motility experiments using individuals with matched and mismatched morphs would provide an interesting test of this adaptive hypothesis.

## 5 Conclusion

Although the role of the elongation of the fourth pair of legs in fighter bulb mites remains unclear, our findings on the dimorphism of these legs and their phenotypic match with the fighting third pair of legs open interesting avenues for future research into the evolution of compensatory traits. Despite the absence of a genetic correlation, the intriguing match between these leg morphs suggests that other mechanisms—beyond developmental integration—may drive the evolution of such traits. Future behavioral experiments on the locomotor performance of fighters and scrambles are needed to determine whether the observed phenotypic correlations between the third and fourth pairs of legs are indeed indicative of compensatory traits. Finally, our study highlights the importance of going beyond simple phenotypic correlations between a weapon and a given structure, as relying solely on these correlations could lead to misinterpretations about the evolutionary role of that structure as a compensatory trait.

## Author Contributions

**Diego Solano‐Brenes:** conceptualization (equal), formal analysis (equal), methodology (equal), writing – original draft (equal), writing – review and editing (equal). **Kyana N. Pike:** data curation (equal), methodology (equal), writing – review and editing (equal). **Joseph L. Tomkins:** data curation (equal), methodology (equal), writing – review and editing (equal). **Glauco Machado:** conceptualization (equal), supervision (equal), writing – original draft (equal), writing – review and editing (equal). **Bruno A. Buzatto:** conceptualization (equal), data curation (equal), formal analysis (equal), methodology (equal), supervision (equal), writing – original draft (equal), writing – review and editing (equal).

## Funding

This work was supported by the Discovery Early Career Researcher Award (DECRA) from the Australian Research Council, DE150101521; Flinders University; Fundação de Amparo à Pesquisa do Estado de São Paulo, 2023/17564‐6; Macquarie University.

## Conflicts of Interest

The authors declare no conflicts of interest.

## Supporting information


**Data S1:** ece372791‐sup‐0001‐Tables.docx.

## Data Availability

The data that support the findings of this study are openly available in figshare at http://doi.org/10.6084/m9.figshare.29794931.
