# Supplementary material for: Phenotypic Matching Without Genetic Correlation in Dimorphic Legs of Bulb Mites
Source: Ecol Evol. 2026 Jan 15;16(1):e72791. doi: 10.1002/ece3.72791 (PMC12808334; doi:10.1002/ece3.72791)
Supplement: Supplementary file 1 — Data S1: ece372791‐sup‐0001‐Tables.docx. [file ECE3-16-e72791-s001.docx]

**SUPPLEMENTARY MATERIAL**

**Table S1** | Linear model results for the effect of body size (anterior coxae suture length), male morph, and their interaction on the length of the fourth pair of legs in fighters of the bulb mite *Rhizoglyphus echinopus*. This model explains approximately 63% of the variability in the length of the fourth pair of legs.

|  | **Estimate** | **Std. Error** | **t-value** | **p-value** |
| --- | --- | --- | --- | --- |
| Intercept (Fighter) | 0.292 | 0.020 | 14.354 | <0.001 |
| Body size | 0.374 | 0.221 | 1.691 | 0.092 |
| Scrambler | -0.113 | 0.028 | -4.090 | <0.001 |
| Body size * Scrambler | 0.570 | 0.305 | 1.870 | 0.062 |

**Table S2** | Heritability estimates, standard errors (SE), and p-values of leg traits in fighters of the bulb mite *Rhizoglyphus echinopus*, based on a half-sibling breeding experiment.

| **Leg traits** | **Heritability** | **SE** | **p-value** |
| --- | --- | --- | --- |
| 3^rd^ leg width | 0.46 | 0.15 | 0.003 |
| 3^rd^ leg length | 0.42 | 0.17 | 0.012 |
| 4^th^ leg width | 0.38 | 0.15 | 0.010 |
| 4^th^ leg length | 0.31 | 0.16 | 0.045 |
